# Expression of Organic Anion Transporting Polypeptide 1c1 and Monocarboxylate Transporter 8 in the Rat Placental Barrier and the Compensatory Response to Thyroid Dysfunction

**DOI:** 10.1371/journal.pone.0096047

**Published:** 2014-04-24

**Authors:** Yi-na Sun, Yuan-jun Liu, Lu Zhang, Yan Ye, Lai-xiang Lin, Yong-mei Li, Yu-qin Yan, Zu-pei Chen

**Affiliations:** 1 Key Laboratory of Hormones and Development (Ministry of Health), Metabolic Diseases Hospital & Tianjin Institute of Endocrinology, Tianjin Medical University, Tianjin, China; 2 Department of Dermatovenereology, Tianjin Medical University General Hospital, Tianjin, China; 3 Science Research Department, Logistics University of the Chinese People's Armed Police Forces, Tianjin, China; Xavier Bichat Medical School, INSERM-CNRS - Université Paris Diderot, France

## Abstract

Thyroid hormones (THs) must pass from mother to fetus for normal fetal development and require the expression of placental TH transporters. We investigate the compensatory effect of placental organic anion transporting polypeptide 1c1 (Oatp1c1) and monocarboxylate transporter 8 (Mct8) on maternal thyroid dysfunction. We describe the expressions of these two transporters in placental barriers and trophoblastic cell populations in euthyroidism and thyroid dysfunction resulting from differential iodine nutrition at gestation day (GD) 16 and 20, that is, before and after the onset of fetal thyroid function. Immunohistochemistry revealed that in the blood-placenta barrier, these two TH transporters were strongly expressed in the villous interstitial substance and were weakly expressed in trophoblast cells. Levels of Oatp1c1 protein obviously increased in the placental fetal portion during maternal thyroid deficiency at GD16. Under maternal thyroid deficiency after the production of endogenous fetal TH, quantitative PCR analysis revealed down-regulation of Oatp1c1 occurred along with up-regulation of Mct8 in trophoblast cell populations isolated by laser capture microdissection (LCM); this was consistent with the protein levels in the fetal portion of the placenta. In addition, decreased D3 mRNA at GD16 and increased D2 mRNA on two gestational days were observed in trophoblast cells with thyroid dysfunction. However, levels of Oatp1c1 mRNA at GD16 and D3 mRNA at GD20 were too low to be detectable in trophoblast cells. In conclusion, placental Oatp1c1 plays an essential compensatory role when the transplacental passage of maternal THs is insufficient at the stage before the fetal TH production. In addition, the coordinated effects of Oatp1c1, Mct8, D2 and D3 in the placental barrier may regulate both transplacental TH passage and the development of trophoblast cells during thyroid dysfunction throughout the pregnancy.

## Introduction

Thyroid hormones (THs) are crucial for fetal growth and development, especially for the central nervous system (CNS). Several studies have shown that early maternal thyroid insufficiency has the potential to impair fetal neurodevelopment [1], [2], [3], [4], [5]; these studies have generally increased scientific interest related to the effects of the transplacental passage of maternal THs on normal fetal CNS development. Evidence shows that maternal THs can cross the placenta while playing a role in modulating fetal-placental development throughout the pregnancy [6]. First, maternally derived TH, notably thyroxine (T_4_), when transported via the placenta, is known to alter neural progenitor proliferation, differentiation, and migration within the developing embryo before the onset of endogenous TH production [3], [5], [7], [8], [9], [10]. Also, evidence shows maternal thyroid dysfunction directly influences placental development itself. *In vitro* studies have demonstrated that THs influence villous and extravillous trophoblast proliferation, viability, and invasive ability [11], [12]. However, scientists still have an incomplete understanding of the molecular mechanisms controlling the transfer of maternal THs across the placental barrier.

Some proteins are reported to be involved in transplacental TH passage. Placental deiodinase D3, and to a lesser extent D2, control intraplacental TH levels. D3 in the villous trophoblast is widely believed to catalyze inner ring deiodination of 3,5,3′-triiodothyronine (T_3_) and T_4_ into biologically inactive metabolites and that D3 plays a key role in protecting the fetus from excessive TH exposure [13], [14]. Also, the activation of the predominant circulating TH, T_4_, into the active ligand, T_3_, by D2 in the villous trophoblast probably provides T_3_ for ‘housekeeping’ processes, and its activity is much less than that of D3 [14].

However, only D2 and D3 regulated the metabolic processes of THs in placental tissues but both do not participate in the entry of THs into cells. The consensus that membrane transporters mediate cellular uptake and efflux of THs [15], [16], has been widely accepted because the discovery of mutations in the TH membrane transporter gene, Mct8, which results in severe neurological impairment [17], [18] suggests this. To date, a range of plasma membrane TH transporters have been identified in human and rodent placental tissue: Mct8, Mct10, Oatp1a2, Oatp4a1, Lat1 and Lat2 [19], [20], [21]. In addition, an important TH transporter with the highest apparent affinity for T_4_, Organic anion transporting polypeptide 1c1 (Oatp1c1 and also known as Slco1c1 and Oatp14), is one of the organic anion transporting polypeptide (OATPs) genes, classified within the solute carrier gene family [22]. Oatp1c1 is mainly expressed in the blood–brain barrier as well as the blood-cerebrospinal fluid barrier and could be essential for thyroid hormone delivery to the developing brain [23]. Nevertheless, we do not know if Oatp1c1 is expressed in the placental barrier and is involved in the transplacental passage of maternal THs. Because transplacental maternal T_4_ is considered important for early brain development during embryogenesis and Oatp1c1 showed the highest apparent affinity for T_4_, we hypothesize that Oatp1c1 is the active plasma membrane TH transporter in rat placenta and plays a critical part in the physiological regulation of maternal to fetal TH transfer, especially during early gestation. Meanwhile, Mct8, which has been reported to be a specific T_3_ transporter in neurons [17], [24], mediates T_3_ uptake in the microvillous plasma membrane of human term syncytiotrophoblasts [25] and makes a significant contribution to TH transport by human placental cells [26]. Therefore, we hypothesize that rat Mct8 participates in transplacental TH transfer from mother to fetus.

Structurally, a placenta forms a barrier; THs from maternal circulation within both human and rodent placenta have to traverse the trophoblast layer, villous stroma, and capillary endothelium before entering fetal circulation. Therefore, this study focused on the villous trophoblast cells of the rat placental barrier, which serves as the first plasma membrane barrier to the transplacental passage of maternal THs. We aimed, before and after the endogenous fetal TH production, to identify the expression of Oatp1c1 and Mct8 in the normal fetal portion of rat placental tissue and the location in the rat placental barrier. And then we assessed the expression of Oatp1c1 and Mct8 in the fetal portion of placenta and trophoblast cells from rat pregnancies complicated by maternal thyroid dysfunction which was resulted from mild to severe iodine deficiency. Therefore, the purpose of this study was to investigate a particular role of placental Oatp1c1 and Mct8 in compensating for a TH shortage.

## Materials and Methods

### Ethics statement

The treatment of all animals in this study followed the guidelines of the Guide for the Care and Use of Laboratory Animals (United States National Institutes of Health). The Laboratory Animal Care Committee of Tianjin Medical University approved all experimental procedures and protocols. All reasonable efforts were made to minimize the number of animals used and their suffering.

### Animals and study design

**Figure 1:**
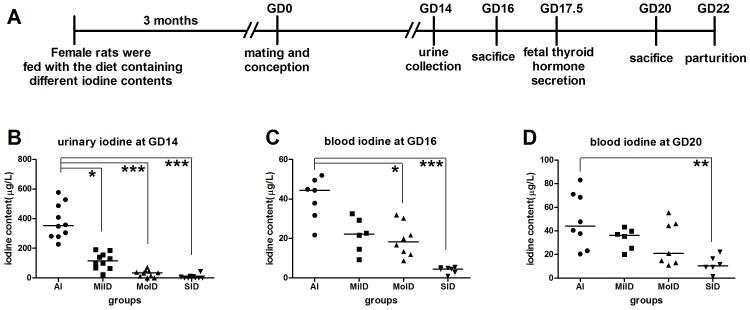
Schematic of experimental timeline (A) and iodine contents in urine (B) and blood(C, D) of pregnant rats. Data are presented as median values. Note that the amplitudes of the various iodine levels were different among the groups as follows: blood iodine < dietary iodine supply < urine iodine. Data are presented as medians (by Kruskal-Wallis test; *p<0.05; **p<0.01; ***p<0.001 vs. same-day AI group)14 days: AI: n = 10, MiID: n = 10, MoID: n = 10, SID: n = 10; 16 days: AI: n = 7, MiID: n = 6, MoID: n = 8, SID: n = 7; 20 days: AI: n = 8, MiID: n = 6, MoID: n = 7, SID: n = 6.

Healthy Wistar rats (Vital River Laboratory Animal Technology, Inc., Beijing, China) 4–6 weeks old and weighing 70–100 g each were housed at 26±3°C, with a light-dark period of 12 h and a relative humidity of 50%. They were fed with normal food (iodine content was 300–400 µg/kg) and allowed free access to tap water (iodine content was 8 µg/L). After acclimatization to the laboratory conditions for 1 week, 64 female rats were randomly divided into four groups: an adequate iodine group (AI or control group), a mild iodine deficiency group (MiID), a moderate iodine deficiency group (MoID), and a severe iodine deficiency group (SID). Iodine-deficient food that was fed to female rats was prepared by our laboratory to reproduce an animal model of iodine deficiency (the foodstuff with an iodine content of 50 µg/kg consisted of 40% soybean, 30% corn, 30% wheat, essential trace elements, and vitamins); rats were given tap drinking water with different doses of potassium iodide until sacrifice (Table 1). Female rats were fed for 3 months before mating. Male rats were fed on normal food and tap water throughout experiment. Fetal thyroid hormone secretion occurs from 17.5 days gestation [27] and parturition at 22 days gestation. Pregnant rats and their placentae at gestational day (GD) 16 and 20 were studied. Figure 1A describes the project timeline.

**Table 1:** Estimated daily iodine intake for maternal rats in four groups.

Groups	Amount of KI added to tap water artificially (µg/L)	Iodine supply in drinking water (µg/day)	Total daily iodine intake (µg/day)
AI	381.7	9.0	10.0
MiID	163.8	4.0	5.0
MoID	54.9	1.5	2.5
SID	0	0.24	1.24

Rats absorb 1 mg of iodine from food each day, in which the iodine intake estimation was based on 20 g food intake each day and 50 µg/kg iodine content in food (50 µg/kg×20 g/day). 0.24 mg iodine was taken by rat from tap water each day, which was based on a daily requirement of 30 mL water and 8 µg iodine content in tap water (8 µg/L×30 mL/day).

### Determination of iodine

Urine of pregnant rats was collected by metabolic cage at GD14. Maternal femoral arterial blood with heparin anticoagulant was collected at GD16 and GD20. Iodine levels in urine and blood were detected by As-Ce catalytic spectrophotometry as previously described [28].

### Hormone measurements

Femoral arterial blood and amniotic fluid (AF) collected from the pregnant rats were allowed to stand for 30 min at room temperature; then they were centrifuged at 3,000 rpm for 15 min. The amniotic fluid sample of per dam was pooled over six fetuses. Thyrotropin (TSH) and THs in serum samples were measured by a chemiluminescent immunoassay technique that we have reported previously [29].

### Western immublots

Fetal sides (labyrinth) separated from placental discs at two ages (GD16 and GD20) were washed with ice-cold 1× phosphate-buffered saline and homogenized by a model DIAX900 homogenizer (Heidolph Elektro, Germany) in RIPA buffer (Cell Signaling Technology, Danvers, MA, USA) containing protease inhibitors for 1 min. Homogenates were incubated on ice for 30 min followed by centrifugation at 14,000 rpm for 10 min at 4°C to precipitate debris. In addition, rat brain and kidney homogenates were obtained in the same way as that of the positive (Oatp1c1 and Mct8) and negative (Oatp1c1) control, respectively [30]. The supernatant was collected and the protein concentration was measured using a BCA Protein Assay Reagent Kit (Pierce Biotechnology, Rockford, IL, USA). 100 µg samples were fractionated by sodium dodecyl sulfate–polyacrylamide gel electrophoresis (10% gel). Protein was transferred onto a polyvinylidene fluoride membrane (Millipore, Billerica, MA, USA) and blocked with 5% skim milk in Tris-buffered saline including 0.1% Tween-20 (TBS-T buffer) for 2 hours at room temperature, and then subjected to immunoblot analysis with antibodies to Oatp1c1 and Mct8 (sc-134802 and sc-47126, 1∶500) as well as β-actin (sc-47778, 1∶2000, Santa Cruz Biotechnology, Inc., Santa Cruz, CA, USA). After over-night incubation at 4°C in primary antibodies, immune complexes were detected with horseradish-peroxidase-conjugated secondary antibodies. Enhanced chemoluminescence was detected using ECL reagent (Millipore). One membrane was incubated with three primary antibodies one by one. The immunoblots using one primary antibody was subsequently stripped and reprobed with the next primary antibody. The relative amounts of specific antibodies present in various samples were estimated by densitometric scanning of the X-ray film and analyzed by the Image System (Gene snap). The data were recorded with the net OD corrected for background chemiluminescence.

To demonstrate the specificity of the Oatp1c1 (sc-134802) and Mct8 (sc-47126) antibodies, we obtained the full-length cDNA clones of rat Oatp1c1 (NM053441) and Mct8 (NM147216) by gene synthesis and cloned them into pcDNA3.1 (GeneWiz, Suzhou, Jiangsu, China) respectively. Then, empty vector (VO), pcDNA3.1-rOatp1c1 or pcDNA3.1-rMct8 was transfected into 293T cells and whole-cell protein extracts were probed with these two antibodies. Antigen absorption was performed by incubating anti-Mct8 antibodies with the blocking peptide (sc-47126 P) (2 µg/mL).

### Immunohistochemistry

Placental tissues were fixed in 4% (w/v) paraformaldehyde overnight and stained with a streptavidin-biotin complex kit (Boster, Wuhan, Hubei, China) according to the manufacturer's instructions. Tissues were incubated with rabbit-anti-rat Oatp1c1 and Mct8 polyclonal antibody (sc-134802 and sc-135156, 1∶50, Santa Cruz Biotechnology) at 4°C overnight, incubated with biotinylated goat-anti-rabbit IgG (Boster) at 37°C for 40 min. Nuclei were counterstained with haematoxylin. Rat cerebrum, as the positive control, was stained with placenta simultaneously. Negative control immunostaining was also performed for brain and placenta sample by omitting the primary antibody. Pictures were taken with an Olympus BX53 microscope (Olympus Corporation, Tokyo, Japan) equipped with a DP72 Microscope Digital Camera and Imafe-Pro Plus 7.0 software.

### Laser capture microdissection

The trophoblast layer of placenta in every group was isolated using laser capture microdissection. A three mm-thick section of central placenta was embedded in OCT medium and 8 µm-thick tissue cryosections were immobilized on SuperFrost PLUS slides and air-dried for 10 min. After re-hydration in diethylpyrocarbonate (DEPC)-treated water, sections were lightly stained with DEPC-treated haematoxylin, and then were kept in a desiccator until capture. Capture was done by Laser Microdissection and Pressure Catapulting using a MicroBeam microscope (P.A.L.M. Microlaser Technologies). Captured material was placed directly into RNA extraction buffer and kept at −80°C until use.

### RT-PCR analysis

Total RNA was extracted from normal frozen fetal parts of rat placenta using TRIzol Reagent (Sangon, Shanghai, China) and LCM samples (two cell populations pooled per sample) using a RNeasy FFPE Kit ((QIAGEN, Valencia, CA, USA) according to the supplier's recommendations. Total RNA of normal rat placenta (2 µg) and of the extracted LCM samples (0.2 µg) was reverse-transcribed to complementary DNA as previously described [29].

### Quantitative real-time PCR (qPCR)

Quantitative realtime PCR was carried out as previously described [29]. Ubiquitin C (UBC) was used as a housekeeping gene for normalization. The following primers were chosen to generate the PCR fragments: Oatp1c1, 5′- GCAAATGTTCAGACTCAAAATGGG -3′ and 5′- ATATAATGTTCTTTCCACTCCGGC -3 [31]; Mct8, 5′- TGGTTACTTCGTCCCCTACG -3′, and 5′- CCAGGGATGGAGTCACTGAT -3′ [32]; D2, 5′- ACTCGGTCATTCTGCTCAAG -3′ and 5′- CAGACACAGCGTAGTCCTTC -3′; D3, 5′- CTCGAACTGGCAACTTTGT -3′ and 5′- GTGAGATGCTGGCGACTTAT -3′; UBC, 5′- TCGTACCTTTCTCACCACAGTATCTAG -3′ and 5′- GAAAACTAAGACACCTCCCCATCA -3′. All primers were observed to produce a PCR product that resolved as a single band with no primer-dimer on agarose gels. All samples were run in triplicate and repeated on another day. The annealing temperature was 60°C for all primer pairs.

The “4–6-point method” was used to calculate Oatp1c1, Mct8, D2, D3 and UBC initial template quantity as described by Ramakers [33]. The ratios of Mct8/UBC, Oatp1c1/UBC, D2/UBC and D3/UBC were used as the initial template relative expression level for Oatp1c1, Mct8, D2 and D3 mRNA, respectively.

### Statistical analysis

Statistical analyses were performed using the SPSS 19 statistical package. Iodine levels were expressed as the median and determined with the nonparametric- Kruskal-Wallis- test. The other quantitative data were expressed as mean ± SD. Differences between groups were evaluated by one-way ANOVA and F-test. If ANOVA showed significant differences between groups, individual groups were compared with the control group by Least Significant Difference or Tamhane's T2-test. In the statistical analyses, a *p* value of <0.05 was considered statistically meaningful.

## Results

### Iodine levels

The iodine levels in urine and blood of pregnant rats decreased as the iodine supply of female rats decreased. However, the extent of decrease in urine iodine level was significantly higher than that of blood iodine level (Figure 1B–D).

### Maternal serum hormone levels

**Figure 2:**
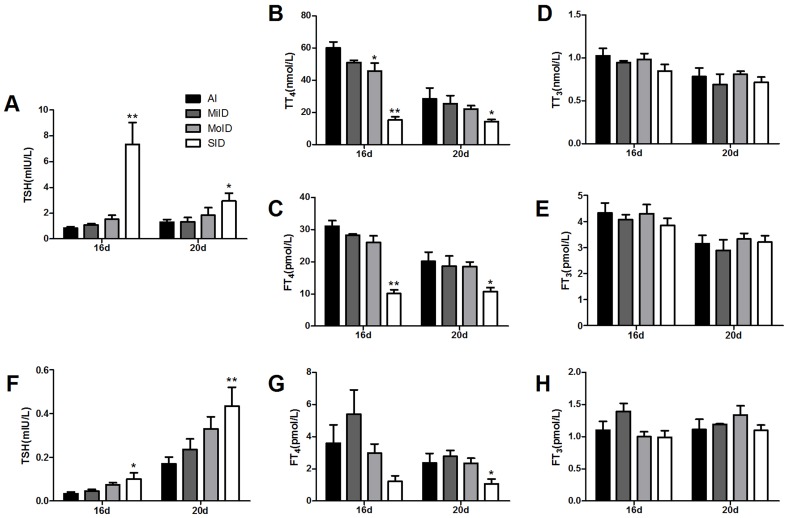
CLIA analysis thyroid hormones levels of dam serum and pup AF at two gestational days (16th and 20th day). Data are presented as mean±SD (by ANOVA; *p<0.05; **p<0.01 vs. same-day AI group). Serum at GD16: AI: n = 8, MiID: n = 7, MoID: n = 8, SID: n = 8; Serum at GD20: AI: n = 8, MiID: n = 7, MoID: n = 7, SID: n = 8; AF at GD16: AI: n = 6, MiID: n = 7, MoID: n = 8, SID: n = 7; AF at GD20: n = 6 in all groups.

Generally, we found that an increase in TSH levels was followed a decrease in total T_4_ (TT_4_) levels among all groups, and TT_4_ and free T_4_ (FT_4_) levels gradually decreased over time at the two ages of pregnancy following a decrease in iodine supply (Figure 2A–E).

Specifically, compared with the AI group, serum TT_4_ and FT_4_ levels decreased significantly and TSH levels increased significantly in pregnant rats of the SID group at GD16 and GD20 (*p*<0.01 and *p*<0.05, respectively). TT_4_ levels in the MoID group decreased significantly (*p*<0.05) compared with those in the AI group at GD16. TT_4_ and FT_4_ levels tended to decrease non-significantly and TSH levels tended to increase non-significantly in the MiID group on two gestational days. However, there were no obvious changes in serum total T_3_ (TT_3_) and free T_3_ (FT_3_) with decreased iodine intake.

### Fetal AF hormone levels

We did not detect any level of TT_4_ and TT_3_ in all AF samples. However our results show that the levels of TSH of AF gradually increased at two gestational days following a decrease dose of iodine supplement, which was consisted with the levels of TSH changes in the dam serum. Compared to the AI group, in the SID group, the TSH levels in AF significantly increased both at GD16 and GD20 (*p*<0.05 and *p*<0.01, respectively) but the FT_4_ levels were only decreased at GD20 (*p*<0.05). Especially, no statistical drop was observed in AF FT_4_ levels of the SID group at GD16. For other experiment groups, the AF FT_4_ levels have no significant changes along all the time points of monitoring. In addition, in all test groups, the FT_3_ levels have no significant changes either (Figure 2F–H).

### Oatp1c1 and Mct8 mRNA and protein levels in the fetal portion of rat placenta

**Figure 3:**
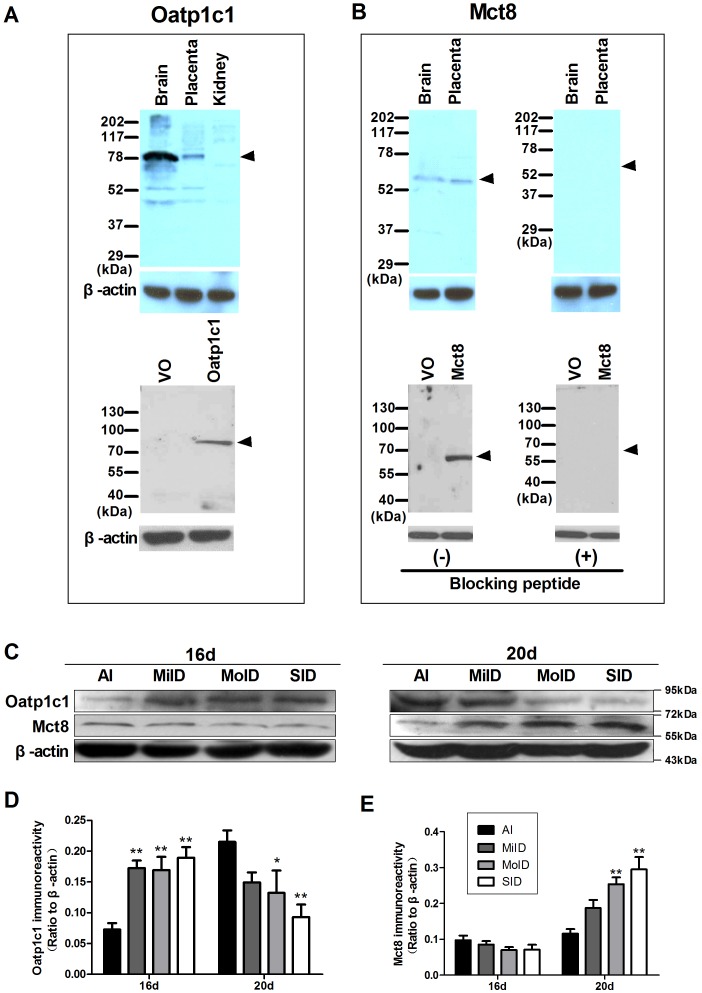
Expression of Oatp1c1 and Mct8 protein levels in the fetal sides of rat placenta. (A) Immunoblotting with Oatp1c1 antibody (sc-134802) using proteins prepared from rat brain (positive control), placenta, kidney (negative control) and 293T cells transfected with either vector only (VO) or a plasmid encoding rat Oatp1c1. (B) Immunoblotting with Mct8 antibody (sc-47126) using proteins prepared from rat brain (positive control), placenta and 293T cells transfected with either vector only or a plasmid encoding rat Mct8 in the absence or presence of Mct8 blocking peptide (sc-47126P). Arrow heads indicate the expected position of the band. (C) Immunoblotting with Oatp1c1 and Mct8 antibodies using placenta protein (100 µg/lane) of all the groups at two gestational stages. Ratios of Oatp1c1/β-actin (D) and Mct8/β-actin (E) immunoreactive densities were determined for each group. β-Actin was used to assess protein loading and sample integrity. For (D) and (E) each bar represents the mean ± SEM of each group (by ANOVA; *p<0.05; **p<0.01 vs. same-day AI group). Three placental discs per litter were pooled one sample and every group had five samples.

Using immunoblotting with the lysate from 293T cells transfected with a plasmid encoding rat Oatp1c1 and the homogenates from rat brain and placenta, anti-Oatp1c1 antibody was recognized an expected band at about 79 kDa, but not with 293T cells transfected with empty vector (VO) and kidney tissue (Figure 3A) which was described in a previous report [30]. In addition, a single band at about 63 kDa was detected with anti-Mct8 antibody in the lysate from 293T cells transfected with a plasmid encoding rat Mct8 and the homogenates from brain and placenta, but not in the 293T cells transfected with empty vector. These bands were absent after pre-absorbing anti-Mct8 antibody with the blocking peptide (Figure 3B).

In the AI group, mRNA and protein levels of Oatp1c1 in the fetal portion increased over time as the pregnancy progressed but those of Mct8 were observed without an obvious increase with age. The mRNA levels at two days of pregnancy (GD16 and GD20) were as follows: Oatp1c1 were 0.66±0.25 (10^−3^) and 2.71±0.61 (10^−3^), and Mct8 were 4.92±0.62 (10^−2^) and 5.20±0.99 (10^−2^), respectively. The protein expression displayed as Figure 3C–E.

In the results of Western blot analysis in the fetal portion of rat placenta at GD16, the level of Oatp1c1 protein expression was obviously higher in the three iodine deficiency groups than that of the AI group (all *p*<0.01). Nevertheless, Mct8 protein level at GD16 was slightly down-regulated in the three iodine deficiency groups, but there was no significant difference compared with the control group (AI).

Interestingly, the protein expression of two transporters in placenta of iodine deficiency groups at GD20 displayed a reverse pattern. Oatp1c1 protein levels showed gradually lower expression with a decrease in iodine intake having a statistical significance in the MoID and SID group compared with the AI group (*p*<0.05 and *p*<0.01, respectively). However, Mct8 protein was gradually up-regulated with the decrease in iodine intake and levels in the MoID and SID groups were significantly higher compared with the AI group (all *p*<0.01).

### Location of Oatp1c1 and Mct8 proteins in rat placental barrier

**Figure 4:**
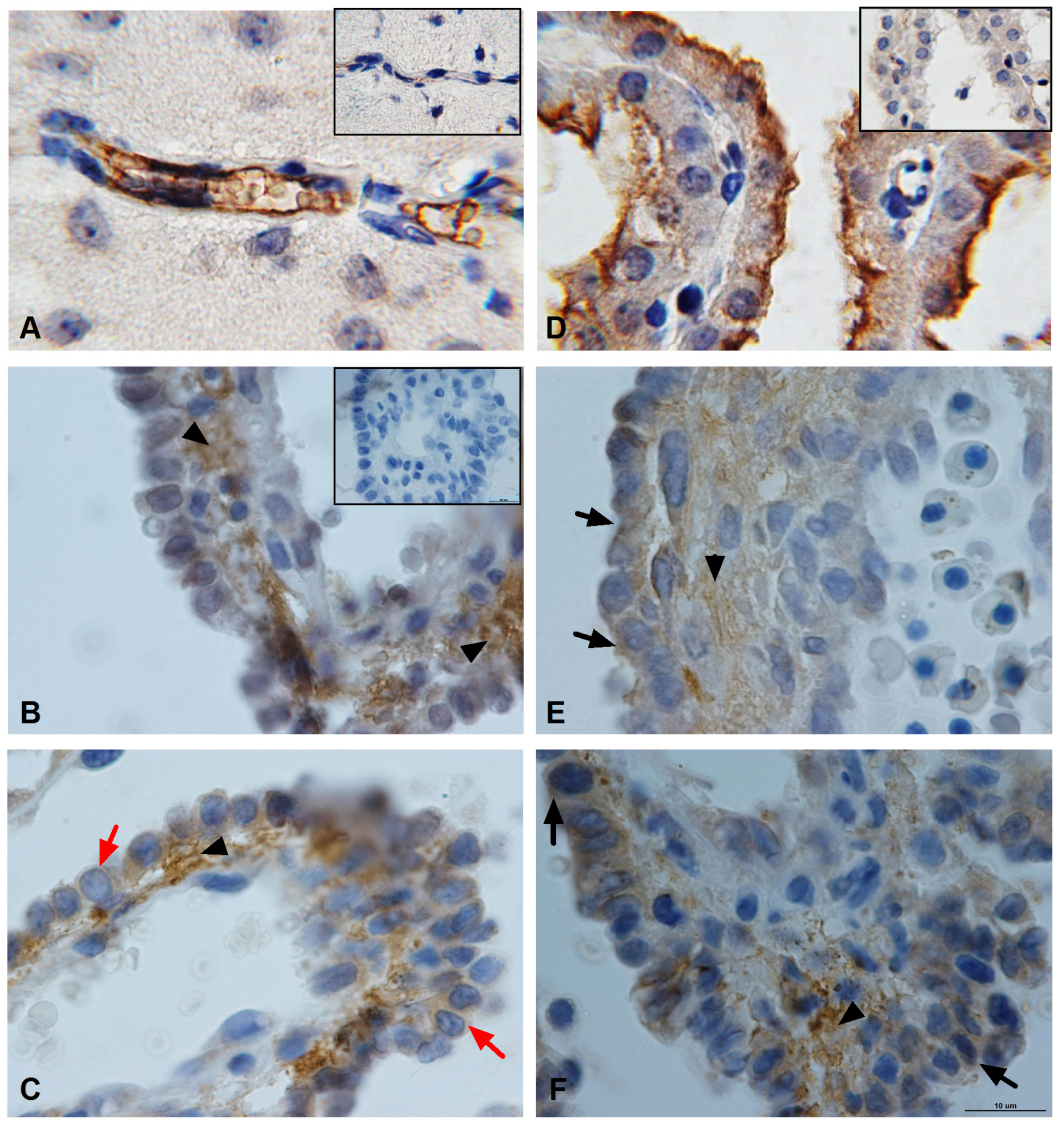
Representative immunohistochemical staining of Oatp1c1 and Mct8 in normal rat placental barrier. The expression and tissue distribution of Oatp1c1 and Mct8 were assessed on fixed placental tissue sections. (A) Oatp1c1 staining using antibody (sc-134802) in the capillary wall of rat cerebral cortex (positive control). (B) and (C) showed Oatp1c1 staining at GD16 and 20, respectively. (D) Mct8 staining using antibody (sc-135156) on the apical surface of rat choroid plexus (positive control). (E) and (F) showed Mct8 staining at GD16 and 20, respectively. In the villous interstitial substance forming the blood-placenta barrier in normal rat placenta, strong staining was seen with both antibodies (labeled by black arrow head ▴). However, Oatp1c1 was poorly observed in trophoblast cells at GD 16 but only weak staining of Oatp1c1 at GD20 (labeled by red arrow) as well as Mct8 at GD16 and 20 were observed on the membrane and in the cytoplasm of trophoblast cells (labeled by black arrow). Corresponding negative controls (PBS instead of anti-Oatp1c1 and anti-Mct8 antibodies) of adjacent sections are shown in panel inserts in the upper right corner for (A, B and D).

We used immunohistochemistry to analyze the location of the Oatp1c1 and Mct8 proteins in rat placental barrier of normal iodine intake. Oatp1c1 and Mct8 immunoreactivities were detected in rat cerebrum as the positive controls. As previously reported [34], [35], both transporter stainings were observed primarily in microvessels and choroid plexus epithelium of rat cerebrum. Figure 4A and 4D show typical pictures of Oatp1c1 staining in the capillary wall and Mct8 staining on the apical surface of rat choroid plexus, respectively, confirming the specificity of staining.

In this study, Oatp1c1 and Mct8 immunoreactivities were mainly present in the villous interstitial substance forming the blood-placenta barrier in rat placenta and their expressions in the villous interstitial substance exceeded by far that of the trophoblast cells (Figure 4 B, C, E, and F). Oatp1c1 was barely observable in trophoblasts at GD16 (Figure 4B) but at GD 20 this protein was weakly detected on the membrane and in the cytoplasm of trophoblast cells (Figure 4C). However, lower levels of Mct8 were present in trophoblasts at both gestational days (Figure 4E and 4F). Nevertheless, both transporters were expressed in trophoblast cells, mostly in the apical side, and were also expressed in the basal side of some cells. In addition, the two transporter location of rat placental barrier in other three experimental groups was consistent with that of the AI group (the figures were not showed here).

### TH transporters and deiodinases mRNA levels in trophoblast cells

**Figure 5:**
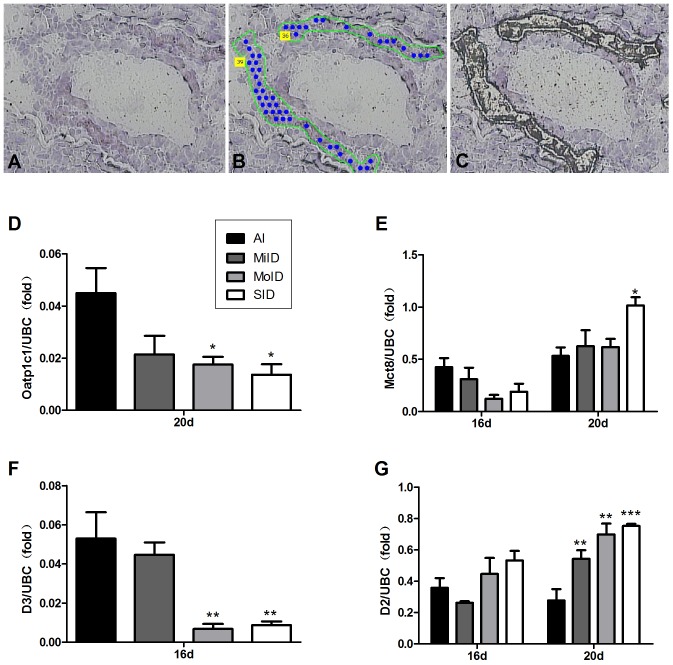
mRNA expressions of TH transporters and deiodinases in the rat placental trophoblast cells acquired by LCM. (A, B, C) Laser capture microdissection of HE-stained trophoblasts from cryosections of rat placental tissue. All photography was performed with the 20X objective. (A) Trophoblasts were selectively identified. (B) Areas of trophoblast-rich populations were cut and captured using the UV cutting laser and capture IR laser, respectively. (C) Selected trophoblasts were removed from the tissue. Oatp1c1 (D), Mct8 (E), D3 (F) and D2 (G) gene expression was normalized with the reference gene UBC. Each bar represents the mean ± SEM of each group (by ANOVA; *p<0.05; **p<0.01; ***p<0.001 vs. same-day AI group). Cells of one litter were isolated from three placental discs and two litters were pooled one sample. Every group had three samples.

Figure 5A–C shows the placental barrier in cryosections and the process used to obtain the trophoblast cells from rat placental tissue by LCM. Figure 5D–G shows the expression of TH transporters and deiodinase mRNA levels in the rat placental trophoblast cells acquired by LCM.

Oatp1c1 mRNA of the trophoblast cells purified by LCM in all groups was too low to be detectable at GD16 by quantitative PCR. However, although the level of Oatp1c1 mRNA was lower at GD20, it was detectable by qPCR in the four groups. Meanwhile, compared with the AI group, the mRNA expression of Oatp1c1 in both the MoID and SID groups significantly decreased (*p*<0.05; Figure 5D).

Mct8 mRNA of the trophoblast cells purified by LCM was detectable at two gestational stages. The increases were observed only in the iodine deficient groups except the AI group with advancing gestation. Expression of Mct8 mRNA had decreased insignificantly in the three iodine deficiency groups at GD16 when compared with control group (Figure 5E). However, at GD20, as trophoblast cells developed the mRNA expression of Mct8 was gradually up-regulated with the decrease of iodine intake. A statistically significant upregulation for Mct8 was observed in the SID group when compared with the AI group (*p*<0.05).

D3 mRNA of the trophoblast cells purified by LCM in all groups was detectable at GD16, but undetectable at GD20 by quantitative PCR. Moreover, its mRNA level at GD16 was reduced with the decrease of iodine intake and there was significant difference in the MoID and SID groups compared with the AI group (all *p*<0.01; Figure 5F).

In the case of iodine deficiency, D2 mRNA in purified trophoblast cells was higher than that in AI group at both GD16 and GD20, except for the MiID group at GD16. The increasing tendency of D2 mRNA levels with the decrease of iodine intake was quite similar at GD16 and GD20. However, compared with control group, no significant difference was found at GD16 but the obvious increase was observed in all experimental groups at GD20 (*p*<0.01 for MiID and MoID and *p*<0.001 for SID; Figure 5G).

## Discussion

This study provides the first report on changes in the rat placental expression of Oatp1c1. These data also provide the first direct evidence that Oatp1c1 and Mct8 are strongly expressed in rat placental villous stroma and show the compensatory mechanisms in pathological situations of either maternal and/or fetal THs deficiency at different stages of gestation *in vivo*.

To date, Oatp1c1 has been reported be expressed at the highest levels in the blood-brain barrier of rat and mouse as well as in many different brain regions and testis Leydig cells of human [36], [37], [38]. In addition, Mct8 is predominantly expressed in the brain and mediates thyroid hormone uptake from the circulation required for normal neural development [39]. Several studies have demonstrated that the expression of multiple TH transporters occurs at the trophoblastic cell barrier [21] and suggested multiple TH transporters are involved in placental T_3_ and T_4_ uptake from maternal blood with no single one of them playing a dominant role [25]. In our study, mRNA encoding Oatp1c1 was expressed in the fetal side of rat placenta and showed a greater than 4-fold increase during later gestation when compared with earlier gestation. Nevertheless, Mct8 mRNA levels in fetal portion and trophoblasts of normal rat placenta were observed without an obvious increase from GD16 to GD20, while in normal human placenta MCT8 mRNA was detected from early in the first trimester and exhibited a significant increase with advancing gestation [21]. Because rather late gestational time points (G16 and G20) were analyzed in this study, the significant increase of rat placental Mct8 mRNA possibly happened before the gestational day 16. Most surprisingly, immunohistochemistry illustrated Oatp1c1 protein was mainly present in the villous stroma at the rat placental barrier; nevertheless, the trophoblasts were barely observable at GD16, but Oatp1c1 protein was weakly stained on the membrane and in the cytoplasm of trophoblast cells at GD20. In addition, Mct8 protein was also present in large quantities in the villous stroma and weakly stained in trophoblast cells of the rat placental barrier.

To further illustrate whether or not the two TH transporters were expressed in normal rat placental trophoblast cells, their mRNA level was detected by qPCR in a trophoblast population collected by LCM. The level of Oatp1c1 was too low to be detectable in trophoblast cells at GD16. However, although the levels of Oatp1c1 mRNA at GD20 and Mct8 mRNA at GD16 and GD20 were very low, they were detectable by qPCR in purified trophoblast cells from the rat placental barrier. The increase of Mct8 mRNA in trophoblasts was insignificant in the AI group with advancing gestation and only was observed in the iodine deficient groups what is, therefore, rather an effect of TH deprivation. We provide the first description of the location of Oatp1c1 and Mct8 protein in villous stroma of the rat placental barrier which was previously unknown [6]; this also implies that Oatp1c1 and Mct8 expression of villous stroma could be important to intraplacental TH transfer and metabolism.

In humans, iodine deficiency during gestation is well known to affect fetal and infant brain development, leading to a decline of mental activity and, in extreme cases, cretinism [40]. Iodine intake increases during pregnancy to meet an increment of about 50% of thyroid hormone production required for fetus development. When compounded by the physiological increases in iodine requirements of pregnancy, therefore, even mild iodine deficiency can result in impaired maternal and fetal production of TH [41]. Hypothyroxinemia (normal TSH with low FT_4_) and subclinical hypothyroidism (elevated TSH with normal free FT_4_) resulting from mild to moderate iodine deficiency is more common than hypothyroidism. Mild iodine deficiency leading to hypothyroxinemia is a much more prevalent throughout the world compared to a classical hypothyroid condition [42].

In our study, for all three experimental groups of dams, daily iodine intake did not meet their physiological requirements for iodine and as a result their urine and blood iodine levels decreased with the reduced iodine intake. However, the decreased levels of the urine iodine were significantly higher than that of blood iodine and iodine was obviously reduced by excretion in urine [29]. Therefore, even though more iodine was retained in the body by compensation from the kidney, blood iodine did not meet the needs of thyroid hormone synthesis in MoID and SID dams. The results indicated that hypothyroidism had occurred in SID dams GD16 and GD20, while hypothyroxinemia occurred in MoID dams at GD16. Although TSH levels, TT_4_ and FT_4_ levels of MoID dames at GD20 and MiID dams at both GD16 and GD20 were not significantly different when compared with the AI group, the mild rise in TSH levels and reduction in TT_4_ and FT_4_ levels showed very slight thyroid dysfunction. No matter what thyroid dysfunction of dams experienced, serum TT_4_ and FT_4_ levels of pregnant rats gradually declined as a result of mild to severe iodine deficiency at both GD16 and GD20.

The thyroid function in dams was clear; however, the thyroid function in pups is still unknown. Detecting levels of TSH and THs in serum proved to be challenging due to the limit volume of serum available from pups. Also, other have reported that human fetal thyroid function can be evaluated by detecting TSH and THs contents in AF [43], [44]. So, we detected the TSH and THs levels in AF of the fetal rats; we found that hypothyroidism and subclinical hypothyroidism were observed in SID fetus at GD20 and GD16, respectively. However, no obvious changes in fetal thyroid function from the mild to moderate experimental groups at these two stages were observed; these findings were different from slight thyroid dysfunction of MiID dams and hypothyroxinemia of MoID dams. Therefore, we deduced that the TH transporters of placental tissue participated in the compensatory regulation in order to maintain the normal thyroid function of fetus when maternal thyroid function was abnormal.

TH transporters with different affinities for specific forms of TH [45], [46], as well as for other compounds which can act as competitive inhibitors (with the exception of MCT8), probably offer flexibility to ensure that the requisite amount of TH is available for fetal-placental development at each stage of gestation. The use of combinations of transporter inhibitors that have an additive effect on TH uptake, therefore, provide evidence that 67% of saturable T_4_ uptake is facilitated by system-L and MCT10 with a minor role played by organic anion-transporting polypeptides, whereas 87% of saturable T_3_ uptake is mediated by MCT8 and MCT10 [25]. However, the factors regulating the placental expression of these transporters are unknown. Some evidence suggests that in rodents the activity of system-L and the expression of Mct8 as well as Oatp1c1 in non-placental tissues are influenced by thyroid status [22], [47], [48], which suggests that TH may be a regulator of its own transporters. Therefore, in pathological situations of either maternal or fetal thyroid hormone deficiency during pregnancy, certain transporters could compensate for a lack of TH in placenta, such as a compensatory up-regulation of MCT8 in placentae from IUGR pregnancies delivered in the early 3rd trimester compared to age matched appropriately grown for gestational age controls [20], [21].

Consequently, our current results demonstrated, in the case of maternal thyroid dysfunction, compensatory up-regulation of Oatp1c1 expression took place in placental homogenate before the onset of fetal TH production while that of Mct8 expression was displayed both in placental homogenate and trophoblastic cells after the onset of fetal TH synthesis. These results provide support for the hypothesis that TH may be a regulator of its own transporters and the Oatp1c1 and Mct8 play an important role in placental TH transfer and metabolism. Also, recent studies made the same conclusion and identified the important effects of Oatp1c1 and Mct8 on brain thyroid hormone metabolism [22], [49]. Considering the expression of placental D2 and D3, we believed there could be a similar pattern of THs metabolic process in placenta and brain, as described below.

The concentration of fetal TH circulating in the blood and fetal brain is closely correlated to levels of maternal free T_4_ rather than free T_3_ [50] and transplacental transport of maternal TH is evidenced by the finding of a biologically significant concentrations of FT_4_ in fetal coelomic fluid and in fetal tissues from as early as five weeks of gestation [51], [52]. As a result, FT_4_ is believed to be the major form of TH transported across the placenta. In early rat placenta before the onset of fetal thyroid function, when Oatp1c1 protein expression has significantly increased, the level of Mct8 protein decreased in maternal thyroid dysfunction compared to maternal euthyroidism. The increasing Oatp1c1 protein improved the transplacental supply of T_4_ and increases the T_4_ concentration in fetal circulation while the decreasing Mct8 expression restricts the transplacental transfer of maternal T_3_ which allows the dams to maintain the T_3_ concentration in maternal circulation itself. Sugiyama D et al [22] also reported the expression levels of Oatp1c1 mRNA and protein in the rat brain up-regulated under hypothyroid conditions. However, no change in the expression and activity of placental D3 in hypothyroid rats or humans has been documented [13], [53], [54]. Our D3 mRNA findings have not reflected this observation; in contrast, to optimize intracellular T_3_ level, a slight up-regulation of D2 promoted the conversion of T_4_ to T_3_ and the down-regulation of D3 inhibited degradation of T_4_ and T_3_ in trophoblastic cells of iodine deficient pregnant rats. Our findings are largely in agreement with previous studies on D2 activity showing its marked up-regulation in the presence of low thyroid hormone levels [55] and on strongly decreased D3 activity [56] in the brain under those conditions. Overall, attempting to increase transplacental maternal-fetal TH transport and to protect the feto-placental unit from lower circulating TH levels in the case of iodine and TH deficiency, a compensatory mechanism in placenta before the onset of fetal thyroid function is largely facilitated by placental Oatp1c1 with a minor role played by D2 and D3. Therefore, the effect of Oatp1c1 regulation on transplacental thyroid hormone, especially T_4_, could be more important to fetal brain development in iodine and TH deficiency during early gestation.

With advancing gestation, in the case of maternal thyroid dysfunction compared to euthyroid controls at GD20, rat placental Oatp1c1 did not significantly increase but was obviously down-regulated, whereas Mct8 protein of fetal side homogenates of placenta and mRNA of trophoblastic population increased suddenly to a very high level. Although the transplacental passage of maternal THs could continue until delivery, athyroidal human fetuses born at term have (maternally derived) circulating T_4_ at concentrations of 25–50% of normal-term fetuses [57], [58]. Since endogenous fetal TH contributes the dominant amount of TH for fetal circulation, therefore, maternally derived T_4_ is not ignored but plays a minor role to fetal development after the onset of fetal thyroid function. So, placental down-regulated expression of Oatp1c1 adapted to the decreasing delivery of maternal T_4_ when mothers suffered from TH deficiency in late pregnancy. However, one very optimistic finding was that the obvious up-regulation of Mct8 expression in rat placenta with TH deficiency could facilitate transplacental TH delivery. In addition, up-expressions of Mct8 and D2 in trophoblastic cell population may reflect a compensatory mechanism where the body is attempting to increase T_3_ uptake by trophoblast cells as previously reported by Loubière LS et al [21].

In summary, our present study demonstrates that Oatp1c1 and Mct8 are present in the rat placenta barrier at two gestational stages. In the case of maternal TH deficiency resulting from decreasing iodine intake, the coordinated effects of Oatp1c1, Mct8, D2 and D3 in specific body parts may regulate both transplacental TH passage from mother to fetus and the development of the trophoblastic cell throughout the pregnancy. Before the onset of fetal thyroid function, in addition to deiodinases, up-expression of Oatp1c1 in placental villous stroma contributed to maternofetal transfer of TH, whereas up-expression of Mct8 in placental villous stroma and trophoblast cells may be part of the pathophysiology of thyroid dysfunction after the onset of fetal thyroid function.
